# The role of etoposide in the treatment of adult patients with hemophagocytic lymphohistiocytosis

**DOI:** 10.1186/s40164-022-00362-2

**Published:** 2023-01-09

**Authors:** Timo C. E. Zondag, Aglina Lika, Jan A. M. van Laar

**Affiliations:** 1 Department of Internal Medicine, Clinical Immunology Section, Erasmus University Medical Center, ‘s-Gravendijkwal 230, 3015 CE Rotterdam, The Netherlands; 2 Department of Biostatistics, Erasmus MC University Medical Center, Rotterdam, The Netherlands; 3 Department of Pediatrics, Center for Lysosomal and Metabolic Diseases, Erasmus MC University Medical Center, Rotterdam, The Netherlands; 4 Department of Epidemiology, Erasmus MC University Medical Center, Rotterdam, The Netherlands; 5 Department of Immunology, Clinical Immunology Section, Erasmus University Medical Center, Rotterdam, The Netherlands

**Keywords:** Hemophagocytic lymphohistiocytosis, Histiocytic disorder, Etoposide, Meta-analysis, Immuno-chemotherapy, HLH-94 criterion, Systematic review

## Abstract

Hemophagocytic lymphohistiocytosis (HLH) is a potentially fatal inflammatory clinical condition, in which an exaggerated immune response is ineffectively regulated. Although etoposide-containing regimens are generally recommended for children with HLH, the exact role of etoposide in the adult setting remains unclear. We performed a systematic review of the literature on the use of etoposide in adults with HLH. All articles written in English on the use of etoposide in adults with HLH available from seven databases and published on or before July 2021 were analyzed. Thirteen studies were found to be relevant to the search results. Ten of these studies report a statistical analysis on the effect of etoposide, of which five found etoposide-containing regimens superior to non-etoposide-containing regimens. Seven studies provided sufficient data to be included in the meta-analysis. For these data, the estimated logit relative risk of etoposide on survival was 1.06 (95% confidence interval: 0.92–1.21, standard error: 2.06). The pooled data of the meta-analysis did thus not support a beneficial effect of etoposide. It should be taken into account that the presented results are highly susceptible to bias and that the effect of etoposide differs between HLH-triggers. Although the meta-analysis does not support the effect of etoposide, we do not recommend abandoning etoposide as treatment modality. The limitations of the meta-analysis, together with several individual articles confirming the benefit of etoposide, justify etoposide for select cases in adults with HLH such as refractory or severe disease with (threatening) multiorgan failure.

**Supplementary Information:**

The online version contains supplementary material available at 10.1186/s40164-022-00362-2.

To the editor,

HLH is a severe and life-threatening immunological dysregulation either caused by genetic mutation (familial HLH; FHL) or secondary to various triggers (secondary HLH; sHLH). The estimated incidence of FHL is 1 to 1.5 per million children per year [1].

Although etoposide-containing regimens are generally recommended for children with HLH, the exact role of etoposide in the adult patient remains unclear. The treatment strategy in adult patients with sHLH, as recommended by the interdisciplinary working group on adult HLH of the Histiocyte Society, does include etoposide in its treatment strategy [2]. Following this recommendation, etoposide could be considered for sHLH with all underlying triggers, although its use in auto-immune and immunotherapy associated HLH is more restricted [2]. However, evidence to support the use of etoposide in adult sHLH patients is scarce. Therefore we performed a systematic literature review and meta-analysis on the clinical use and effectiveness of etoposide in adult HLH patients. A detailed description of the methods including the search strategy is available in the Additional file 1.

The seven studies that are included in the meta-analysis (Table 1) show an estimated logit relative risk (RR_L_) of 1.06 (standard error: 2.06; 95% CI: 0.92–1.21) (Fig. 1). The survival probability of the etoposide-treated patients did thus not differ significantly from the survival probability of the non-etoposide-treated patients. As detailed in the Additional file 1, the homogeneity was not rejected. Five individual studies show an analysis that is significantly in favor of etoposide [3–7] whereas five other papers report no additional benefit of etoposide [8–12] (Table 1). Similar to a study by Imashuku et al. [13] Song et al. [6] also analyzed patients receiving etoposide within 4 weeks after diagnosis and compared this group with a group of patients receiving etoposide 4 weeks after diagnosis or who did not receive etoposide. No significant difference was observed in survival between the two groups (p = 0.163).

**Table 1:** Articles reporting the effect of etoposide in adults with hemophagocytic lymphohistiocytosis

Author	Year	Reference	Trigger	Total number of adults	Inclusion in meta–analysis	Survival of etoposide–treated patients, % (n)	Survival of non–etoposide–treated patients, % (n)	Additional information	Supporting the effect of etoposide	Risk of bias according to ROBINS–I
Song et al.	2019	[15]	Pregnancy	13	Yes	100% (6)	71% (5)		NS	Critical
Knaak et al.	2020	[16]	Various	40	Yes	14% (1)	45% (15)		NS	Critical
Naymagon et al.	2021	[12]	Various	90	Yes	21% (9)	33% (16)	Log–rank test for difference in the survival distribution (p = 0.41)	No	Critical
Diack et al.	2020	[8]	Various	26	Yes	29% (2)	26% (5)	p = 0.9	No	Critical
Ahn et al.	2010	[17]	Various	26	Yes	31% (4)	69% (9)		NS	Critical
Barba et al.	2015	[9]	Various	71	Yes	54% (15)	67% (29)	p = 0.3	No	Critical
Arca et al.	2015	[3]	Various	162	Yes	85% (69)	74% (60)	p = 0.079, aOR: 0.21, p = 0.04	Yes	Serious
Bigenwald et al.	2018	[4]	Malignancy	71	No			uHR: 0.55 (p = 0.04), aHR: 0.50 (p = 0.04)	Yes	Critical
Bubik et al.	2020	[5]	Various	31	No			HR: 0.22 for ≥ 5 doses of etoposide (p = 0.003)	Yes	Critical
Li et al.	2020	[7]	B–cell lymphoma	31	No			Log–rank test for difference in survival distribution (p = 0.0183)	Yes	Critical
Song et al.	2019	[6]	EBV	58	No			Etoposide as 1st line therapy vs. no etoposide or 2nd line therapy (p = < 0.001)	Yes	Critical
Buyse et al.	2010	[10]	Various	56	No			EIT for non–survivors 6 h vs. survivors 4 h (p = 0.19)	No	Serious
Schram et al.	2015	[11]	Various	68	No			OS etoposide: 9.5 months, OS no etoposide: 1.9 months (p = 0.78)	No	Critical

*aHR* adjusted hazard ratio, *aOR* adjusted odds ratio, *EBV* Epstein–Barr virus, *EIT* etoposide initiation time (time from intensive care unit admission to etoposide initiation), *HR* hazard ratio, *NS* not stated, *OS* overall survival, *uHR* unadjusted hazard ratio

**Fig. 1:**
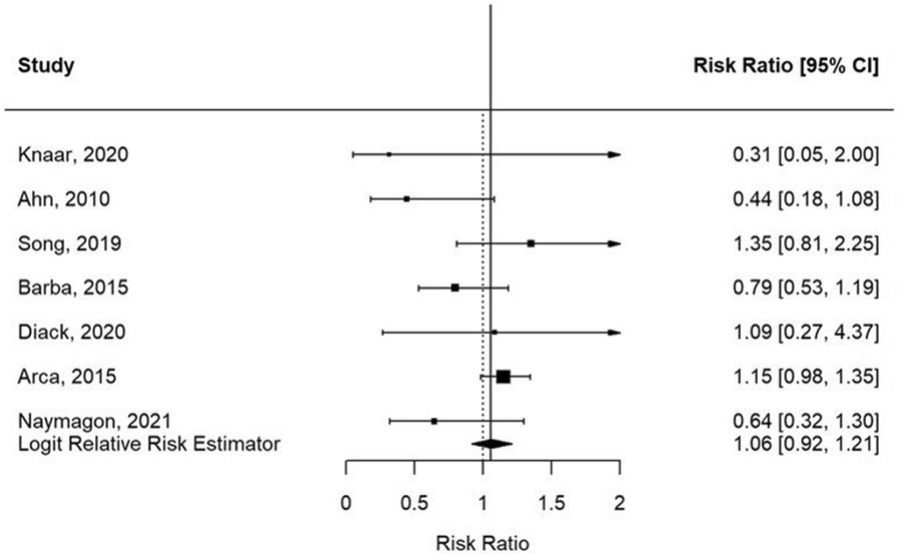
The estimated relative risks of seven studies, which provided data on an etoposide-treated group and a non-etoposide-treated group. The black vertical line represents the logit relative risk estimator. *CI* confidence interval

The presented results should be interpreted with caution. All studies concern retrospective cohort studies and used different statistical methods. In our meta-analysis we used the risk ratio for addressing the outcome. Due to a lack of provided data by the articles, we could not use a more suitable time-to-event measure such as a hazard ratio. Moreover, there is a high risk of bias in all studies (Table 1). In particular, confounding by indication should be noted since patients receiving etoposide generally concern more severe cases and consequently have a prior survival probability which is lower. As the confounding by indication is in favor of non-etoposide-treated patients, a stronger benefit of etoposide than the calculated effect size could be assumed.

The seven studies included in the meta-analysis were homogeneous based on the findings of the *χ*^2^ homogeneity test (Additional file 1). However, within individual groups (i.e. etoposide and non-etoposide-treated), a high degree of heterogeneity is assumed to be present. For example, the studies included patients with diverse etiological HLH triggers, all having a different a priori survival rate [14]. Etoposide may have a different effect among patients with these different etiological triggers. Moreover, several confounders are assumed to effect outcome and should ideally be taken into account. Therefore, it is highly favorable to perform an alternative/additional analysis taking (baseline) confounders into account. In this regard, it would be of particular interest to sub-analyze groups by HLH trigger, since our data suggests that etoposide might be especially beneficial in EBV and lymphoma associated HLH (Table 1) [4, 6, 7]. Owing to the lack of data, we were unable to perform such analysis. However, assuming an equal degree of heterogeneity among the groups (i.e. etoposide and non-etoposide-treated), the data will be averaged out and will thus bring forward a pooled data set that might be compared, although with caution. Given the available data, we believe that this approach is the best available method to address the research question but we also emphasize its limitation.

It is important to note that the studies included in the meta-analysis primarily concern studies that do not present data that support the effect of etoposide (one out of seven studies showing benefit, Table 1). On the contrary, the studies that are not included in the meta-analysis primarily concern studies that do show a benefit of etoposide (four out of seven studies showing benefit, Table 1). Only taking the meta-analysis into account might thus underestimate the effect of etoposide.

The data presented by the meta-analysis should not lead to abandoning etoposide as a treatment modality. The limitations of the meta-analysis that generally lead to an underestimation of the effect size of etoposide, together with several individual articles confirming the benefit of etoposide, justify etoposide for individualized cases of adult HLH. These data support the recent management recommendations by the interdisciplinary working group on adult HLH of the histiocyte society [2]. According to this guideline, it is proposed to initiate a monitored step-up approach starting with corticosteroids and IVIG, especially in patients with mild or moderate disease. Etoposide can be considered for individualized treatment of cases of refractory or severe disease with (threatening) multiorgan failure.

Conclusive studies on etoposide as a treatment modality in adults are not available. To make definitive conclusions on etoposide and its timely administration, a collaboration between HLH treatment centers is needed to initiate a prospective randomized controlled trial. Currently, no definite evidence is available to guide which HLH patients may benefit from etoposide. Thus, etoposide should be administered after careful consideration.

## Supplementary Information

**Additional file 1. Fig. S1.** Flow diagram showing the study section process. Additional sections.

## Data Availability

All data generated or analyzed during this study are included in this published article.
