# Supplementary material for: The role of etoposide in the treatment of adult patients with hemophagocytic lymphohistiocytosis
Source: Exp Hematol Oncol. 2023 Jan 9;12:2. doi: 10.1186/s40164-022-00362-2 (PMC9827679; doi:10.1186/s40164-022-00362-2)
Supplement: Supplementary file 1 — Additional file 1. Fig. S1. Flow diagram showing the study section process. Additional sections. [file 40164_2022_362_MOESM1_ESM.docx]

# Additional file 1

## Additional file 1 methods

A systematic review was performed to analyze published articles on the clinical use and effectiveness of etoposide in adults with HLH. English articles published in seven databases (Embase, MedLine, Cochrane Database of Systematic Reviews, Web of Science, Scopus, and Google Scholar) up to July 2021 were searched (search terms displayed below). The titles and abstracts of the studies derived from the search were screened for potentially relevant studies. The inclusion criteria for studies were as follows: a) studies including patients that fulfill 5 out of 8 diagnostic criteria presented by the HLH-04 study, with the disease clinically confirmed by more than one clinician or with an HScore ≥169 (corresponding to 93% and 86% sensitivity and specificity, respectively);(27, 29, 30) b) studies including patients aged 18 years and older at the time of diagnosis; and c) studies including at least five patients treated with etoposide and five patients treated without etoposide. Conference abstracts were excluded because of the lack of peer review, possible double publication, and incomplete data. Additionally, patients treated with stem cell transplantation were excluded to avoid cointervention bias. Studies with a mixed adult and pediatric population were included only if the data on adults could be extracted separately. The full text of the articles derived from the first screening on title and abstract was assessed independently by two authors to judge its relevance. Any discrepancy between the authors’ findings was discussed, after which disagreements were settled. Additionally, the reference lists of the relevant studies were searched for additional publications that have been missed by our search. All eligible articles were assessed for their risk of bias using the ROBIN-I tool, which is developed for the purpose of non-randomized studies of the effects of interventions.(1)

The logit relative risk (RRL) estimator was calculated, and the χ2 homogeneity test was performed to compare the survival rates among the different studies.(31-33) If the critical value of the Chi-square distribution was greater than the observed value of the homogeneity test, then the null hypothesis was not rejected, and we concluded that the survival distribution was the same across the studies. The studies were then considered valid for the meta-analysis and the calculation of the RRL estimator.

## Additional file 1 results

The titles and abstracts of a total of 1705 studies were screened. Twelve studies were found to be relevant after a full-text review. One additional study was included after a reference search (Figure S1). These 13 studies included 743 patients. All studies were retrospective cohort studies, and most studies did not focus specifically on the effectiveness of etoposide. Most studies did not distinguish between different etiological triggers associated with HLH. The statistical methods varied widely among the studies.

Seven out of the 13 studies provided sufficient data for inclusion in the meta-analysis. The homogeneity for these seven studies was not rejected (X2 = 0.31 < $\chi_{6, 0.95}^{2}$ = 12.59), indicating that the distribution of the relative risks of the studies did not differ significantly. We observed that the RRL estimator was included in all 95% of CIs, indicating that the relative risks of the studies did not differ significantly from the RRL estimator. Finally, we observed that the value 1 was also included in the 95% CI of the relative risks of all the seven studies, indicating that the survival probability of a person administered etoposide did not differ significantly from the survival probability of a person not administered etoposide in these studies.

Ten studies statistically analyzed the effect of etoposide. Five of these found etoposide-containing regimens superior to non-etoposide-containing regimens (Table 1). The remaining five studies did not show etoposide to have a beneficial effect.

Arca *et al*.(2) reported survival rates of 85% and 74% in etoposide-treated and non-etoposide-treated patients, respectively. Based on univariate analysis, this difference was not statistically significant (p = 0.079). However, multivariable logistic regression analysis found an odds ratio of 0.21, which was significantly in favor of etoposide (p = 0.04). Song *et al*.(3) focused on the effects of etoposide in Epstein–Barr virus-triggered HLH. This study compared a group receiving etoposide as first-line therapy with a group receiving etoposide as second-line therapy or who did not receive etoposide. A strong benefit was observed on the prognosis with etoposide as first-line therapy (p <0.001). Bigenwald *et al*.(4) reported the survival in a cohort of patients with HLH triggered by malignancy. Both the adjusted and unadjusted analyses found significantly better survival in the patients treated with etoposide (HRs of 0.55 and 0.50, respectively; p = 0.04 in both analyses). Bubik *et al*.(5) described a cohort of patients who received ≥5 doses of etoposide compared with a cohort of patients who received <5 doses. In this cohort, the HR of 0.22 was in favor of the group receiving ≥5 doses of etoposide. Li *et al*.(6) retrospectively studied B-cell lymphoma-triggered HLH. In their study, a Kaplan–Meier curve of OS indicated that etoposide regimens significantly increased survival compared with etoposide-free regimens (p = 0.018).

Naymagon *et al*.(7) conducted a retrospective cohort study of 90 adult patients with HLH with different triggers. The primary focus of the study was the difference in the survival of the etoposide-treated patients versus the non-etoposide-treated patients. The baseline characteristics showed a higher rate and dosage of corticosteroids in the etoposide group than in the non-etoposide group, indicating that the etoposide group had more severe clinical disease than the non-etoposide group. There was no significant difference in survival between the two groups in the unadjusted analysis (log-rank test, p = 0.41) or the multivariable Cox regression analysis (hazard ratio [HR]: 1.067, p = 0.8084). The analysis of subgroups of different etiological HLH triggers did not show any significant benefit in favor of etoposide. Buyse *et al.*(8) analyzed survival according to the time from intensive care unit admission to etoposide initiation. Although not significant, the time to etoposide initiation was shorter in survivors than in non-survivors. Schram *et al.*(9) reported the OS in a multicenter retrospective cohort. Although the OS was longer in the etoposide-treated group than in the non-etoposide-treated group (9.5 months versus 1.9 months), the difference was not statistically significant (p = 0.78).

## Additional file 1 figures

Fig. S1 Flow diagram showing the study section process


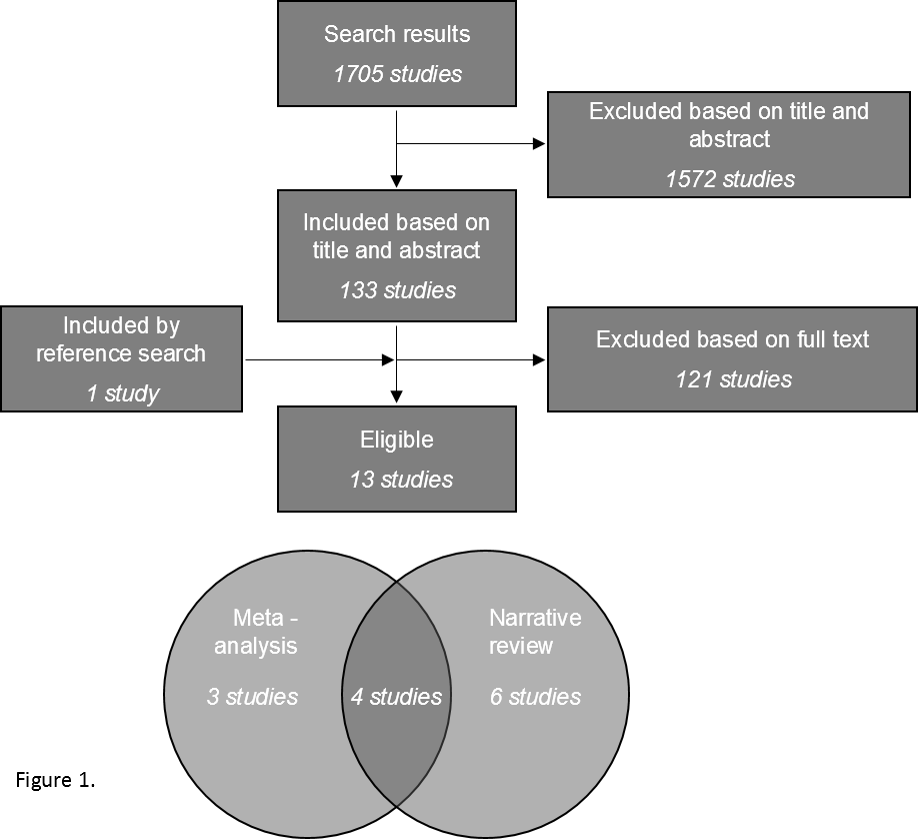


## Additional file 1 search strategy

**Embase.com**

(erythrophagocytosis/de OR 'hemophagocytic syndrome'/exp OR ((phagocytosis/exp OR histiocytosis/exp) AND ('blood cell'/exp)) OR (erythrophag* OR erythrofag* OR hemophag* OR hemofag* OR haemophag* OR haemofag* OR ((erythro* OR hemo* OR haemo* OR leukocyt*) NEAR/3 (phag* OR histiocyto*)) OR (macrophage* NEAR/3 activat*)):ab,ti,kw) AND (etoposide/de OR 'etoposide derivative'/de OR (etoposide* OR celltop OR citodox OR eposin OR epsidox OR etomedac OR etomedec OR etophos OR etopol OR etopos OR etoposid OR etoposido OR etopoxan OR etosid OR lastet OR lastet-s OR nexvep OR nk-171 OR nk171 OR nsc-141540 OR nsc141540 OR posid OR toposar OR topresid OR vepesid OR vepeside OR vespid OR vp-tec OR vp-16 OR vp-16213 OR vp16 OR vp16-213 OR vp16213):ab,ti,kw) AND (adult/exp OR (adult* OR elderl* OR senior* OR older OR (year-old NEXT/1 (female* OR male* OR man OR woman OR men OR women)) OR ((female* OR male* OR man OR woman OR men OR women) NEXT/1 aged)):ab,ti,kw)

**MedLine (OvidSP)**

("Lymphohistiocytosis, Hemophagocytic"/ OR ((exp phagocytosis/ OR exp histiocytosis/) AND (exp "blood cells"/)) OR (erythrophag* OR erythrofag* OR hemophag* OR hemofag* OR haemophag* OR haemofag* OR ((erythro* OR hemo* OR haemo* OR leukocyt*) ADJ3 (phag* OR histiocyto*)) OR (macrophage* ADJ3 activat*)).ab,ti,kf.) AND (etoposide/ OR (etoposide* OR celltop OR citodox OR eposin OR epsidox OR etomedac OR etomedec OR etophos OR etopol OR etopos OR etoposid OR etoposido OR etopoxan OR etosid OR lastet OR lastet-s OR nexvep OR nk-171 OR nk171 OR nsc-141540 OR nsc141540 OR posid OR toposar OR topresid OR vepesid OR vepeside OR vespid OR vp-tec OR vp-16 OR vp-16213 OR vp16 OR vp16-213 OR vp16213).ab,ti,kf.) AND (exp adult/ OR (adult* OR elderl* OR senior* OR older OR (year-old ADJ (female* OR male* OR man OR woman OR men OR women)) OR ((female* OR male* OR man OR woman OR men OR women) ADJ aged)).ab,ti,kf.)

**Cochrane Database of Systematic Reviews**

((erythrophag* OR erythrofag* OR hemophag* OR hemofag* OR haemophag* OR haemofag* OR ((erythro* OR hemo* OR haemo* OR leukocyt*) NEAR/3 (phag* OR histiocyto*)) OR (macrophage* NEAR/3 activat*)):ab,ti,kw) AND ((etoposide* OR celltop OR citodox OR eposin OR epsidox OR etomedac OR etomedec OR etophos OR etopol OR etopos OR etoposid OR etoposido OR etopoxan OR etosid OR lastet OR lastet NEXT s OR nexvep OR nk NEXT 171 OR nk171 OR nsc NEXT 141540 OR nsc141540 OR posid OR toposar OR topresid OR vepesid OR vepeside OR vespid OR vp NEXT tec OR vp NEXT 16 OR vp NEXT 16213 OR vp16 OR vp16 NEXT 213 OR vp16213):ab,ti) AND ((adult* OR elderl* OR senior* OR older OR (year NEXT old NEXT/1 (female* OR male* OR man OR woman OR men OR women)) OR ((female* OR male* OR man OR woman OR men OR women) NEXT/1 aged)):ab,ti,kw)

**Web of Science**

TS=(((erythrophag* OR erythrofag* OR hemophag* OR hemofag* OR haemophag* OR haemofag* OR ((erythro* OR hemo* OR haemo* OR leukocyt*) NEAR/3 (phag* OR histiocyto*)) OR (macrophage* NEAR/3 activat*))) AND ((etoposide* OR celltop OR citodox OR eposin OR epsidox OR etomedac OR etomedec OR etophos OR etopol OR etopos OR etoposid OR etoposido OR etopoxan OR etosid OR lastet OR lastet-s OR nexvep OR nk-171 OR nk171 OR nsc-141540 OR nsc141540 OR posid OR toposar OR topresid OR vepesid OR vepeside OR vespid OR vp-tec OR vp-16 OR vp-16213 OR vp16 OR vp16-213 OR vp16213)) AND ((adult* OR elderl* OR senior* OR older OR (year-old NEAR/1 (female* OR male* OR man OR woman OR men OR women)) OR ((female* OR male* OR man OR woman OR men OR women) NEAR/1 aged))))

**Scopus**

TITLE-ABS-KEY(((erythrophag* OR erythrofag* OR hemophag* OR hemofag* OR haemophag* OR haemofag* OR ((erythro* OR hemo* OR haemo* OR leukocyt*) W/3 (phag* OR histiocyto*)) OR (macrophage* W/3 activat*))) AND ((etoposide* OR celltop OR citodox OR eposin OR epsidox OR etomedac OR etomedec OR etophos OR etopol OR etopos OR etoposid OR etoposido OR etopoxan OR etosid OR lastet OR lastet-s OR nexvep OR nk-171 OR nk171 OR nsc-141540 OR nsc141540 OR posid OR toposar OR topresid OR vepesid OR vepeside OR vespid OR vp-tec OR vp-16 OR vp-16213 OR vp16 OR vp16-213 OR vp16213)) AND ((adult* OR elderl* OR senior* OR older OR (year-old W/1 (female* OR male* OR man OR woman OR men OR women)) OR ((female* OR male* OR man OR woman OR men OR women) W/1 aged))))

**Google Scholar**

Erythrophagocytosis|hemophagocytic|hemophagocytosis|haemophagocytic|haemophagocytosis etoposide adult|adults

## Additional file 1 references

1. Sterne JA, Hernan MA, Reeves BC, Savovic J, Berkman ND, Viswanathan M, et al. ROBINS-I: a tool for assessing risk of bias in non-randomised studies of interventions. BMJ. 2016;355:i4919.

2. Arca M, Fardet L, Galicier L, Riviere S, Marzac C, Aumont C, et al. Prognostic factors of early death in a cohort of 162 adult haemophagocytic syndrome: impact of triggering disease and early treatment with etoposide. Br J Haematol. 2015;168(1):63-8.

3. Song Y, Wang Y, Wang Z. Requirement for etoposide in the initial treatment of Epstein-Barr virus-associated haemophagocytic lymphohistiocytosis. Br J Haematol. 2019;186(5):717-23.

4. Bigenwald C, Fardet L, Coppo P, Meignin V, Lazure T, Fabiani B, et al. A comprehensive analysis of Lymphoma-associated haemophagocytic syndrome in a large French multicentre cohort detects some clues to improve prognosis. Br J Haematol. 2018;183(1):68-75.

5. Bubik RJ, Barth DM, Hook C, Wolf RC, Muth JM, Mara K, et al. Clinical outcomes of adults with hemophagocytic lymphohistiocytosis treated with the HLH-04 protocol: a retrospective analysis. Leuk Lymphoma. 2020;61(7):1592-600.

6. Li B, Guo J, Li T, Gu J, Zeng C, Xiao M, et al. Clinical characteristics of hemophagocytic lymphohistiocytosis associated with non-hodgkin B-cell lymphoma: a multicenter retrospective study. Clin Lymphoma Myeloma Leuk. 2021;21(2):e198-e205.

7. Naymagon L, Tremblay D, Mascarenhas J. The efficacy of etoposide-based therapy in adult secondary hemophagocytic lymphohistiocytosis. Acta Haematol. 2021;144(5):560-8.

8. Buyse S, Teixeira L, Galicier L, Mariotte E, Lemiale V, Seguin A, et al. Critical care management of patients with hemophagocytic lymphohistiocytosis. Intensive Care Med. 2010;36(10):1695-702.

9. Schram AM, Comstock P, Campo M, Gorovets D, Mullally A, Bodio K, et al. Haemophagocytic lymphohistiocytosis in adults: a multicentre case series over 7 years. Br J Haematol. 2016;172(3):412-9.
